# Phytochemicals Attenuating Aberrant Activation of β-Catenin in Cancer Cells

**DOI:** 10.1371/journal.pone.0050508

**Published:** 2012-12-03

**Authors:** Dan Wang, Mitchell L. Wise, Feng Li, Moul Dey

**Affiliations:** 1 Department of Health and Nutritional Sciences, South Dakota State University, Brookings, South Dakota, United States of America; 2 USDA, Cereal Crops Research, Madison, Wisconsin, United States of America; 3 Department of Biology & Microbiology, South Dakota State University, Brookings, South Dakota, United States of America; 4 Department of Veterinary and Biomedical Sciences, South Dakota State University, Brookings, South Dakota, United States of America; George Mason University, United States of America

## Abstract

Phytochemicals are a rich source of chemoprevention agents but their effects on modulating the Wnt/β-catenin signaling pathway have remained largely uninvestigated. Aberrantly activated Wnt signaling can result in the abnormal stabilization of β-catenin, a key causative step in a broad spectrum of cancers. Here we report the modulation of lithium chloride-activated canonical Wnt/β-catenin signaling by phytochemicals that have antioxidant, anti-inflammatory or chemopreventive properties. The compounds were first screened with a cervical cancer-derived stable Wnt signaling reporter HeLa cell line. Positive hits were subsequently evaluated for β-catenin degradation, suppression of β-catenin nuclear localization and down-regulation of downstream oncogenic targets of Wnt/β-catenin pathway. Our study shows a novel degradation path of β-catenin protein in HeLa cells by Avenanthramide 2p (a polyphenol) and Triptolide (a diterpene triepoxide), respectively from oats and a Chinese medicinal plant. The findings present Avenanthramide 2p as a potential chemopreventive dietary compound that merits further study using *in vivo* models of cancers; they also provide a new perspective on the mechanism of action of Triptolide.

## Introduction

Members of the Wnt family of secreted growth factors play important roles during embryogenesis by regulating proliferation, migration, tissue polarity, and organogenesis, and contribute to the development of the genitourinary system. In the canonical Wnt pathway, β-catenin acts as the central component [1]. The binding of Wnt to its receptor (frizzled) and co-receptor low density lipoprotein (LRP5/6) inhibits the formation of the protein complex that includes axin, glycogen synthase kinase-3 (GSK-3), and the adenomatous polyposis coli (APC). This inhibition results in the accumulation of β-catenin in the cytoplasm which subsequently translocates to the nucleus [2]. In the nucleus β-catenin binds to the T-cell factor (TCF) leading to transcription of Wnt target genes [3]. This aberrant β-catenin signaling has been observed in a variety of human cancers including a majority of colorectal cancers, about half of prostate cancers and a third of melanomas [4]. ***The β-catenin also accelerates human papilloma virus type-16 mediated cervical carcinogenesis in transgenic mice*** [***5***] ***.*** Intriguingly β-catenin is thought to influence the metastatic potential of tumor cells by affecting chromatin remodeling [6] as well as modulate oxidative stress response in cells [7]. Hence, attenuation of constitutive activation of the β-catenin signaling pathway has become an attractive target for anti-cancer therapy and prevention.

Naturally occurring compounds represent attractive candidates for development as chemopreventive agents when backed by evidence-based findings and mechanistic research. In particular, dietary agents circumvent the need for additional introduction of foreign compounds into healthy asymptomatic individuals. Dietary agents are also in general less toxic, more readily bioavailable, more accessible, and less expensive as compared to synthetic agents. In the past few years, several laboratories have identified numerous phytochemicals that have potentially useful biological properties; however, only a few groups have directly evaluated the ability of these agents to disrupt β-catenin-mediated Wnt signaling [4]. In this study we report the modulation of lithium chloride (LiCl)-activated Wnt/β-catenin signaling by phytochemicals with known antioxidant, anti-inflammatory and chemopreventive properties. It is proposed that chronic persistent inflammation is a causative factor for a variety of human cancers. While eradication and/or vaccination are reasonable strategies, such efforts can fail to prevent cancer in cases involving persistent inflammation with tissue reorganization and epigenetic changes. Because up-regulation of Wnt family ligands and down-regulation of endogenous Wnt inhibitors occur during the early stage of carcinogenesis, anti-inflammatory agents with the potential to inhibit the canonical Wnt signaling pathway are candidate agents for chemoprevention [8].

Avenanthramides (Avns) are a group of naturally occurring polyphenols found uniquely, among food crops, in oats, a popular healthful cereal [9], [10]. Avn 2p, 2f, and 2c are three major forms that have been intensively studied and are shown to possess superior anti-irritant and antioxidant properties [11], [12], [13], [14]. Bioavailability of Avns has been established in animals and humans [11], [15]. The three Avn congeners appear to be taken up and distributed among the tissues differentially. The rank order of plasma concentration by Avn by type is 2p>2f >2c [16]. Inhibition of *in vitro* colon cancer cell proliferation and NFκB activation in endothelial cells by these compounds has been demonstrated [9], [17]. Here we investigated the potential *in vitro* antiproliferative activity and cellular mechanisms of Avns in human cervical cancer cells.

Phenethylisothiocyanate (PEITC) is a well-studied isothiocyanate, occurring naturally in the form of its glucosinolate precursor, gluconasturtiin, in the Brassicaceae family of vegetables such as cabbage, cauliflower, watercress and broccoli. PEITC showed chemopreventive potential against various cancers [18] and no apparent toxicity even when administered in high doses in rats and dogs as determined by NOEL (no-observed-adverse-effect-level) in drug safety studies [19]. We reported its anti-inflammatory effects and associated cellular mechanisms [20], [21]. It is also proposed that PEITC can suppress cancer metastasis [22]. Since β-catenin is thought to influence the metastatic potential of tumor cells [6], we evaluated any potential effect of PEITC on β-catenin signaling.

Triptolide, a diterpene triepoxide, is a major active component of extracts derived from the medicinal plant *Tripterygium wilfordii* Hook F. Triptolide has multiple pharmacological activities including anti-inflammatory, immune modulation, antiproliferative and pro-apoptotic activity [23], [24], [25]. Triptolide has been widely used in millennia-old medical traditions practiced in China for inflammatory and autoimmune diseases, and more recently for organ transplantation and tumors [26]. Triptolide can induce tumor cell apoptosis directly, as well as enhance apoptosis induced by cytotoxic agents such as TNF-α, and chemotherapeutic agents by inhibiting NFκB activation. Recently, the cellular targets of Triptolide, such as MAP kinase phosphatase-1, Heat shock protein, 5-Lipooxygenase, RNA polymerase and histone methyl-transferases, had been demonstrated [23]. However, to the best of our knowledge, this well-studied and widely used compound has not been evaluated in β-catenin signaling. Hence, we included Triptolide in our current study.

Overall objective for the current study was to establish mechanistic insights of the selected phytocompounds related to Wnt/β-catenin signaling that may be relevant to a broad range of cancers. All experiments were conducted using HeLa cells of human cervical cancer origin (ATCC, Manassas, VA). HeLa cells are co-infected with Papilloma viruses that contribute to a persistent inflammatory response within the cells. Papilloma viruses are thought to be causative factors for human cervical cancers. The HeLa genome has been remarkably stable after years of continuous cultivation; therefore, the genetic alterations detected may have been present in the primary tumor and reflect events that are relevant to the development of cervical and other forms of human cancer [27]. A stable reporter cell line expressing Firefly luciferase under the influence of TCF promoter was produced and optimized to monitor transcriptional activation of the β-catenin/TCF complex in response to intervention with the test compounds. Follow-up investigations included effects of the compounds on cell proliferation, cytosolic β-catenin degradation, nuclear β-catenin localization and downstream expression of c-Myc transcription factor. The Myc proto-oncogene encodes the c-Myc transcription factor, mutation of which contributes to the genesis of many human cancers. As one of the key downstream targets of β-catenin-TCF complex, c-Myc plays a key role in altered cellular metabolism during tumorigenesis [28]. For all experiments, β-catenin stabilization and canonical Wnt signaling were activated using LiCl. GSK-3 is a kinase that phosphorylates β-catenin in the cytoplasm resulting in induction of the canonical Wnt signaling [29]. LiCl activates the canonical Wnt/β-catenin pathway by inhibiting GSK-3 [30], [31], which is independent of any receptor/co-receptor activity. FH535 (Sigma Chemicals, St. Louis, MO) is a small molecule inhibitor of Wnt/β-catenin signaling [32], [33]. FH535 was used in all experiments as a known Wnt inhibitor.

## Materials and Methods

### Plasmids, Chemicals, Cell Lines, and Antibodies

Plasmids 7TFP (Addgene plasmid 24308), pCMVdR8.2 (Addgene plasmid 8455), and pCMV-VSV-G (Addgene plasmid 8454) were purchased from Addgene (Cambridge, MA). The p7TFP vector [34] is a replication incompetent, self-inactivating lentiviral vector containing the 7XTCF promoter, the Firefly luciferase reporter gene and the puromycin resistance cassette for selection [34]. Plasmid pCMV dR8.2 [35] and pCMV-VSV-G [35], encoding VSV-G envelope protein, were used as the lentiviral packing system for producing infectious 7XTFP lentiviruses. The pGL4.75 vector expressing Renilla luciferase gene was purchased from Promega Inc. (Madison, WI). Triptolide, FH535, PEITC, LiCl, Hoechst and polybrene were obtained from Sigma-Aldrich (St. Louis, MO). Puromycin, other antibiotics and cell culture media were purchased from Invitrogen (Grand Island, NY). Reagents used in quantitative PCR, including enzymes were supplied by Applied Biosystems (Foster City, CA). HeLa cell line (CCL-2) and HEK293 T cell line (CRL-11268) were purchased from American Type Culture Collection (ATCC, Manassas, VA). Anti human β-catenin and anti human β-actin antibodies were purchased from Millipore (Billerica, MA). Dylight 680 anti-mouse and Dylight 800 anti-rabbit secondary antibodies were obtained from Li-Cor Biosciences (Lincoln, NE).

### Chemical Synthesis of Avns 2p, 2f and 2c from Oats

Avns 2p, 2f and 2c were synthesized by methods reported previously [36]. Briefly, 5 mmole of the appropriate phenylpropanoid (*p*-coumaric, ferulic or caffeic acid) in pyridine was acetylated with excess acetic anhydride. After reaction, cold water was added and the precipitant was collected and dried overnight. To the acetylated phenylpropanoid in dimethyl formamide, with a small amount of triethylamine, was added, dropwise, an equimolar amount of benzotriazol-1-yloxy)tris-(dimethylamino)phosphonium hexafluorophosphate dissolved in CH_2_Cl_2_. Next 5-hydroxy anthranilic acid (equimolar) in dimethyl formamide was added dropwise. The reaction was stopped with 0.5 M HCl. After extraction of the acetoxyavenanthramide into ethyl acetate and rotary evaporation to dryness, the protecting acetyl groups were removed by addition of 5% pyrollidine in CH_2_Cl_2_. The de-protection reaction was quenched with 1 M HCl and the Avn extracted into ethyl acetate. Purification of the Avn was effected by LH-20 column chromatography.

### Cell Culture

The human cervical cancer cell line HeLa (ATCC, CCL-2), and human embryonic kidney 293T cells (ATCC, CRL-11268) were maintained in Dulbecco’s modified Eagle’s medium supplemented with 100 U/ml penicillin, 100 µg/ml streptomycin, and 10% heat-inactivated fetal bovine serum. HeLa 7TFP stable reporter cell line was maintained in Dulbecco’s modified Eagle’s medium supplemented with 100 U/ml penicillin, 100 µg/ml streptomycin, 0.5 µg/ml puromycin and 10% heat-inactivated fetal bovine serum. The cells were kept in a 37°C incubator with 5% CO2. Cells were subcultured after trypsinization when 90% confluent with a 1∶5 splitting ratio. For cell proliferation assay only, cells were cultured in Dulbecco’s modified Eagle’s medium supplemented with 100 U/ml penicillin, 100 µg/ml streptomycin, 5% heat-inactivated fetal bovine serum, and 50 µM 2-mercaptoethanol. Cells were seeded at different densities as described in each experiment. Viable cell counts were carried out by trypan blue staining using a hemocytometer.

### Lentiviral Production and Stable Reporter Cell Line Development

Lentiviral production was carried out following a modified protocol from Steward *et. al.* [35]. We used a HeLa cell line stably carrying lentiviral 7TFP reporter cassette containing a Firefly luciferase reporter gene controlled by TCF gene promoter [34]. The lentiviral delivered cassette also contains a SV40 promoter-driven puromycin resistance gene [34]. VSV-G pseudotyped lentivirus expressing 7TFP were produced in HEK293T cells by co-transfection of pCMV VSV-G, p7TFP, and pCMV dR8.2. HeLa cells infected with the lentiviruses were incubated in puromycin for 2 wk. Cells surviving antibiotic selection were propagated and characterized for Wnt reporter activity. The lentiviral transduced HeLa 7TFP stable reporter cells were stable for Wnt reporter activity in at least 20 passages (data not shown) when maintained in culture medium containing 0.5 µg/ml puromycin.

### Wnt Reporter Assay

A dual luciferase assay was used to determine the extent of Wnt signaling activation by measuring β-catenin mediated TCF transcription in HeLa 7TFP stable reporter cells in response to treatments with the compounds. First, transient transfection was performed as previously described [37]. HeLa 7TFP reporter cells were grown on 6-cm culture dishes (2×10^6^ cells each dish). After 1d, for each dish, 1 µg of pGL4.75 was mixed with transfection solution containing 3 µl TransIT LT1 (Mirus Bio LLC, Madison, WI) and added to the culture. At 24 h post transfection, cells were trypsinized, resuspended and seeded into wells of a 96-well plate (2×10^4^ cells per well). Six h later, media was replaced with new media containing treatments or vehicle- dimethyl sulfoxide (DMSO). Following 4 h incubation, 50 mM LiCl was added to each well and incubated for an additional 8 h.

The Wnt activation in the various samples was determined by measurement of Firefly luciferase activity using a Dual Glo luciferase assay kit (Promega, Madison, WI). Firefly luciferase activity was measured as luminescence using a Synergy H4 hybrid Reader (BioTek, Winooski, VT) in cell extracts, normalized for Renilla luciferase activity, and expressed as percent change as compared to a LiCl treated positive control. Concentrations of the test compounds and their treatment incubation periods were either pre-determined using separate optimization experiments (data not shown) or obtained from previous reports [9], [20], [24], [32]. Significance of the compound activities were compared on a one-on-one basis with respect to the LiCl control (positive control). Cells treated with vehicle (DMSO only) and not activated using LiCl served as a negative control for normalization of background signal. The data were expressed as percent Wnt transcription reporter activity compared with LiCl.

### Cell Proliferation Assay

The ability of HeLa cells to proliferate in response to various treatments were measured using CellTiter 96® AQueous One Solution Cell Proliferation Assay (Promega Inc., Madison, WI), a colorimetric method for determining the number of viable cells in proliferation. The assay utilizes a tetrazolium compound [3-(4,5-dimethylthiazol-2-yl)-5-(3-carboxymethoxyphenyl)-2-(4-sulfophenyl)-2H-tetrazolium, inner salt; MTS] and an electron coupling reagent (phenazine ethosulfate; PES). Manufacturer’s instruction was followed and treatments were compared to vehicle control (DMSO-treated cells) by reading absorbance at 490 nm on a BioTek Synergy H4 multimode plate reader (BioTek, Winooski, VT). All data shown are means±S.E. from four independent experiments.

### Immunoblotting

HeLa cells (CCL-2, ATCC, Manassas, VA) were seeded into 6 well plates at a density of 1×10^6^ each well. The next day, media was removed and new media containing indicated concentrations of test compounds (Avn 2p, FH535, or Triptolide) or DMSO (vehicle control) were added back. After 4 h, LiCl at a final concentration of 50 mM was added to wells for an additional 8 h incubation. Immunoblotting was carried out following a protocol described by *Gao et. al.* [38]. Briefly, cells were lysed in ice-cold RIPA [150 mM NaCl, 50 mM Tris pH 8.0, 0.4 mM EDTA, 10% glycerol, 1% Nonident P-40 (NP-40)], followed by brief vortexing and rotation for 30 min at 4°C. The supernatant was transferred, and cleared by centrifugation. Equal amounts (v/v) of cell lysates were separated by SDS-PAGE, transferred to nitrocellulose, blocked and double-probed overnight with mouse anti human β-catenin (1∶2000 v/v) and rabbit anti human β -actin (1∶5000 v/v) antibodies, and then incubated with Dylight 680 anti-mouse (1∶5000 v/v) and Dylight 800 anti-rabbit (1∶5000 v/v) antibodies. Blots were imaged with a LI-COR Odyssey Infrared Imaging System (LI-COR Biosciences, Lincoln, NE).

### Immunofluorescence and Nuclear Localization Assay

HeLa cells were seeded into a 12 well plate at a density of 2×10^5^ each well. The next day, culture media was removed and an indicated concentration of each compound (Avn 2p, FH535, or Triptolide) or DMSO was added back. The optimized concentration of each compound was pre-determined by the Wnt reporter assay. After 4 h incubation, 50 mM LiCl was added to wells for an additional 8 h incubation. Immunofluorescence was measured as described previously [37]. Cells were immunostained with mouse anti β-catenin (1∶1000 v/v) and goat anti mouse Dylight 488 (1∶2000 v/v) antibodies sequentially. Nuclei were stained with Hoechst dye (0.1 µg/ml). Images were collected on ***an EVOS FL epifluorescent microscope (AMG, Bothell, WA)*** *.* The imaging data were expressed as Dylight 488 (indicating β-catenin localization), Hoechst (indicating nucleus), and Merge (overlay of Dylight 488 and Hoechst staining for the same view). Nucleus localization of β-catenin was determined by Dylight 488 staining in the nucleus. The cells with presence of β-catenin in the nucleus were counted among 100 cells for each treatment.

### Total RNA Extraction, Purification, and cDNA Synthesis

Total RNA extraction, purification and cDNA synthesis were performed as described by Dey *et. al*. previously [21]. Total RNA was extracted from HeLa cells using TRIzol reagent (Invitrogen, Grand Island, NY) following the manufacturer’s instructions. RNA was then treated with DNaseI (Invitrogen, Grand Island, NY) to remove any traces of DNA contamination according to the manufacturer’s guidelines. RNA was quantified spectrophotometrically by absorption measurements at 260 and 280 nm using the Nanodrop 2000 system (Thermo Fisher Scientifics, Waltham, MA). The cDNAs were synthesized using 3 µg of RNA for each sample using MultiScribe Reverse Transcriptase (Applied Biosystems, Foster City, CA), following the manufacturer’s protocol.

### Real Time Quantitative PCR

Real time quantitative PCR was performed as previously described [21]. The synthesized cDNAs were diluted 2.5-fold. Two microliters of each diluted sample were added to 0.5 µl gene-specific primers (6 µM; oligos synthesized by IDT Inc., Coralville, IA) and 12.5 µl of Brilliant SYBR green PCR master mix (2X) (Applied Biosystems, Foster City, CA). ROX was used as an internal dye. To avoid interference due to genomic DNA contamination, only intron-overlapping primers were selected using the Primer Express version 2.0 software (Applied Biosystems, Foster City, CA) as follows (forward and reverse pairs of the primers are indicated as “F” and “R”, respectively): c-Myc (Genbank ID: NM_002467 ), F: 5′-caccagcagcgactctga-3′, R: 5′-gatccagactctgaccttttgc-3′; β-actin (Genbank ID: NM_007393), F: 5′-ccaaccgcgagaagatga-3′, R: 5′-ccagaggcgtacagggatag-3′. PCR amplifications were performed on a MX3005p system (Stratagene, Santa Clara, CA). A non template control was included in the PCR program as a quality control step. Standard ΔΔCt method was used for relative mRNA quantity calculation with respect to the LiCl-elicited control (positive control) which is normalized to a value of 1.0 as described by Dey *et. al*. [21]. A value of less than 1.0 indicates transcriptional down-regulation (inhibition of gene expression) compared with the LiCl positive control, which shows maximum genetic induction (1.0). Therefore, lower values indicate greater Wnt inhibitory activity. Amplification of specific transcripts was further confirmed by obtaining melting curve profiles. All samples were run in duplicate.

### Statistical Analysis

**Figure 1:**
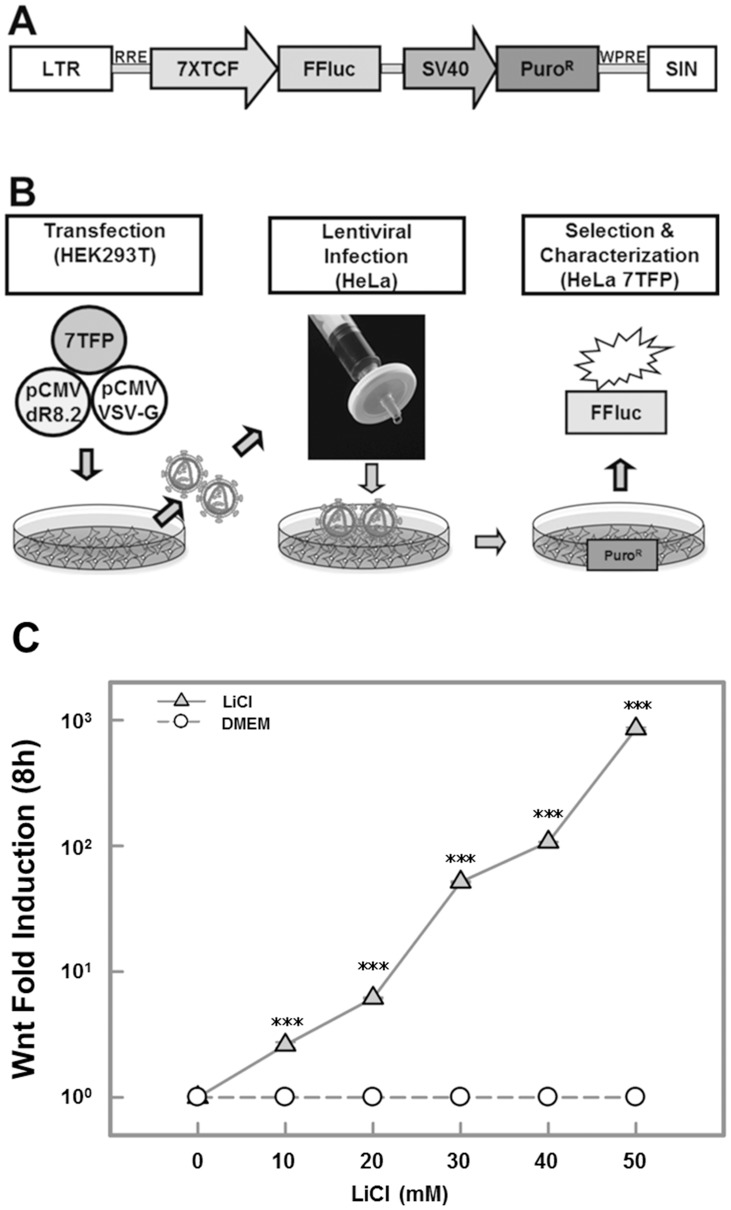
Production of lentiviral induced stable Hela 7TFP Wnt reporter cell line. A. Lentiviral vector 7TFP contains the 7XTCF promoter, the Firefly luciferase, and the puromycin resistance gene under the control of SV40 promoter. LTR, long terminal repeat; RRE, Rev responsive element; FFluc, Firefly luciferase; SV40, Simian vacuolating virus 40; PuroR, puromycin resistance; WPRE, woodchuck hepatitis virus posttranscriptional regulatory element; SIN; self inactivating. B. Schematic representations of the 7TFP lentiviral production by transfection, lentiviral infection, and stable reporter cell line selection. C. Validation of concentration-dependent Wnt reporter response from the stable producer cell line following LiCl induction (8 h) was measured using Luciferase activity. Folds of Wnt reporter activity relative to negative control (vehicle-treated) are shown as mean±S.E.

**Figure 2:**
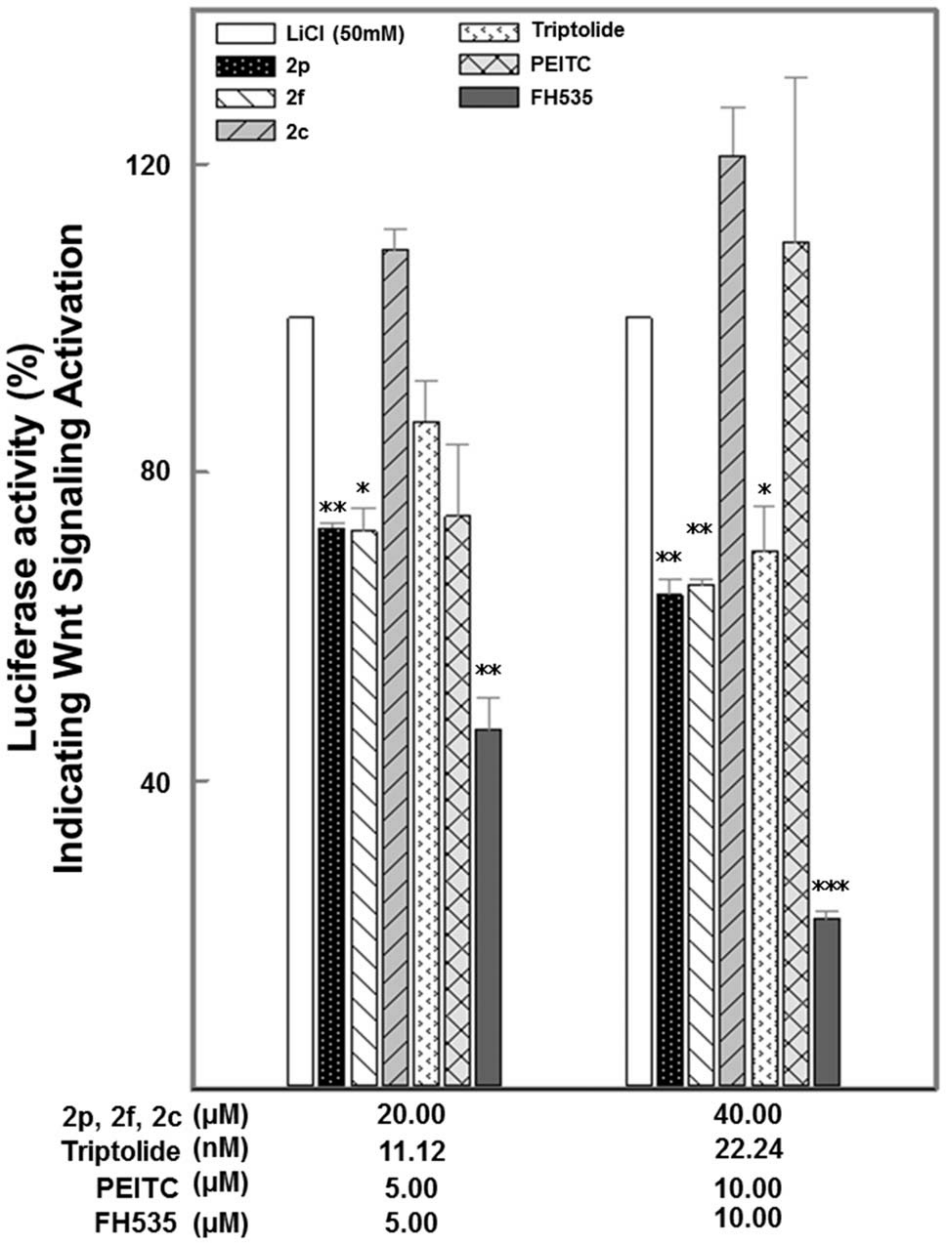
Attenuation of Wnt transcription by phytochemicals in HeLa 7TFP reporter cells. Reporter and Renilla luciferase activities were measured using a Dual Glo luciferase assay kit. Normalized percentage values relative to LiCl control are shown as the mean±S.E (n = 4). Asterisks indicate statistically significant differences between compound*-*treated cells and LiCl-induced control: ***p≤0.001, **p≤0.01, * p≤0.05.

**Figure 3:**
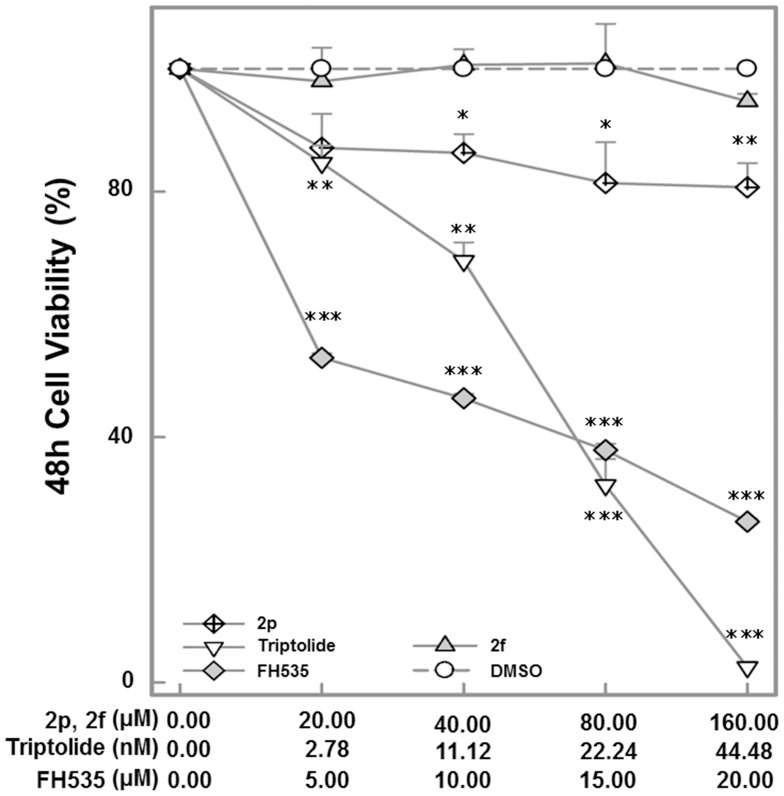
Avn 2p and Triptolide defer HeLa cell proliferation. Hela cells were cultured with MTS culture medium. Absorbance was read at 490 nm and data were expressed as percent cell viability compared with DMSO control (vehicle). Normalized percentage values relative to DMSO control are shown as the mean±S.E (n = 4). Asterisks indicate statistically significant differences between compound*-*treated cells and control: ***p≤0.001, **p≤0.01, * p≤0.05.

**Figure 4:**
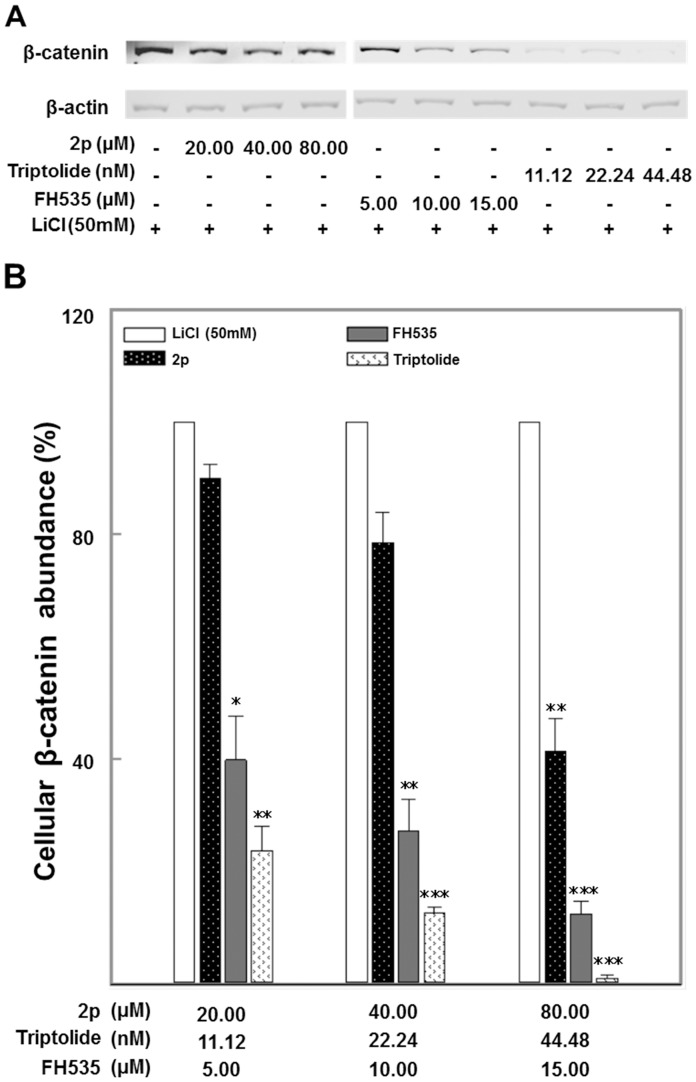
Avn 2p and Triptolide induced cellular degradation of β-catenin. A. β-catenin protein expression: Western blot was double probed with mouse anti human β-catenin (1∶2000 v/v) and rabbit anti human β-actin (1∶5000 v/v) antibodies, washed in phosphate buffered saline, and further incubated with Dylight 680 anti-mouse (1∶5000 v/v) and Dylight 800 anti-rabbit (1∶5000 v/v) antibodies. LI-COR odyssey Infrared Imaging System was used in immunoblotting signal detection. B. Densitometric analysis of β-catenin protein levels (mean±SE, n = 3): β-actin was used as housekeeping control for normalization. Asterisk indicates expression levels of β-catenin in compounds-treated cells that are significantly different from that of LiCl induced control: ***p≤0.001, **p≤0.01, * p≤0.05.

**Figure 5:**
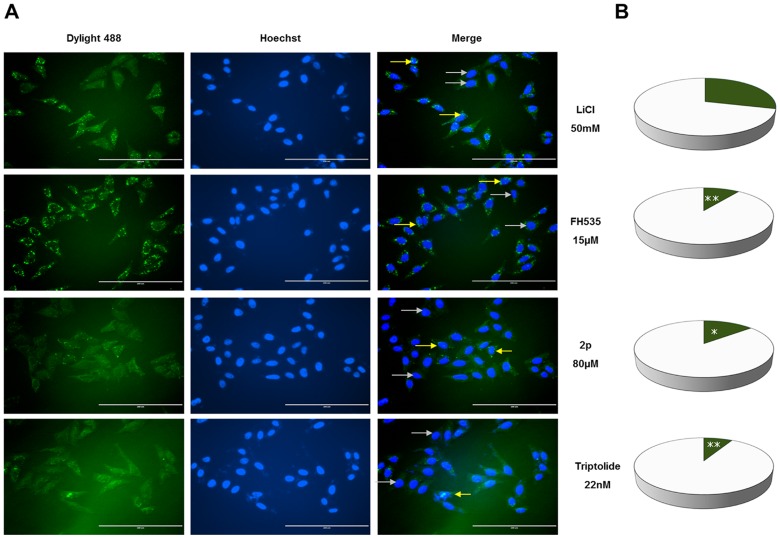
Avn 2p and Triptolide reduced nucleus abundance of β-catenin in HeLa cells. A. Microscopic detection of β-catenin localization by immunofluorescence: HeLa cells were treated with vehicle only (DMSO, top panel), 15 µM FH535 (second from top), 80 µM Avn 2p (third from top) or 22 nM Triptolide (bottom) prior to 50 mM LiCl induction for each. Cells were immunostained with mouse anti β-catenin (1∶1000 v/v) and goat anti mouse Dylight 488 (1∶2000 v/v) antibodies (green), and Hoechst (0.1 µg/ml, blue). Yellow arrows indicate the presence of β-catenin in the nucleus whereas white arrows point to the absence of β-catenin in the nucleus. B. Statistical analysis of β-catenin nucleus localization. Nucleus localization of β-catenin was determined by Dylight 488 staining in the nucleus. The cells with presence of β-catenin in the nucleus were counted among 100 cells for each treatment. Number of cells exhibiting the nuclear translocation of β-catenin is shown in dark green, while number of cells not showing β-catenin’s nuclear localization is shown in white. Asterisks indicate statistically significant differences between compound*-*treated cells and control: ***p≤0.001, **p≤0.01, * p≤0.05, n = 3.

**Figure 6:**
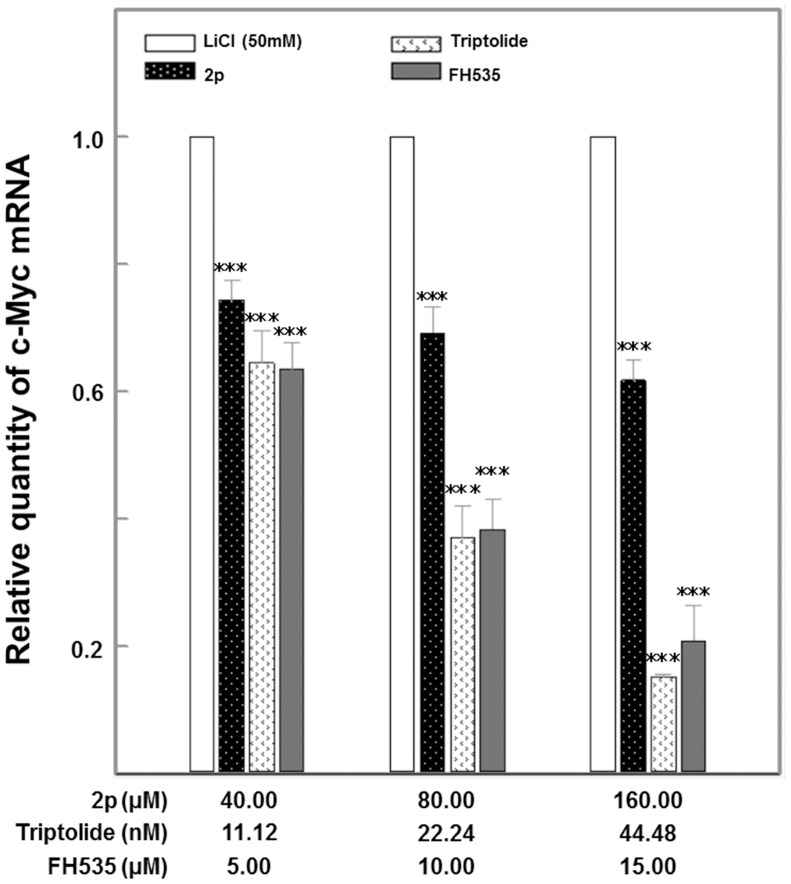
Avn 2p and Triptolide inhibit c-Myc transcription in LiCl-elicited HeLa cells. The concentration-dependent effects of treatments on c-Myc gene expression was measured by the mRNA quantity relative to the response to LiCl activation only (positive control) that is normalized to a value of 1.00; lower values represent greater inhibitory effects with 0.00 corresponding to a complete inhibition of the induced gene expression. Total RNA was extracted, purified and cDNA was synthesized. Relative quantification using SYBR green technology and standard ΔΔCt method was used for individual RT-PCR. Relative values are mean±S.E (n = 3). Asterisks indicate statistically significant differences between compound*-* treated cells and control: ***p≤0.001, **p≤0.01, * p≤0.05.

Experimental observations are expressed as the mean ±S.E. In all figures, the significance of any treatment over the untreated control (DMEM for Fig. 1C, LiCl for Fig. 2, DMSO for Fig. 3, and LiCl for Figs. 4B, 5B and 6) was determined by Student’s *t* test. Treatments were considered significantly different if p<0.05 *, highly significant if p<0.01 * *, or extremely significant if p<0.001 * * *.

## Results

### Production and Optimization of Stable Reporter HeLa 7TFP Cell Line for High Content Screening of Phytochemicals for Anticancer Properties

To identify naturally occurring chemical modulators of Wnt signaling, we developed a high-content assay to measure activation, nuclear translocation and subsequent binding of β-catenin to the TCF transcriptional complex in HeLa cells. Inhibition of the GSK-3 complex in the cytoplasm leads to production of aberrant β-catenin which subsequently translocates to the nucleus to bind to the TCF-complex triggering downstream activation of Wnt pathway genes. We used a HeLa cell line stably carrying a lentiviral 7TFP reporter cassette containing a Firefly luciferase reporter gene controlled by the TCF gene promoter (Fig. 1A). To demonstrate functional significance of the HeLa 7TFP reporter cell line, we used LiCl, a pharmacological inhibitor of GSK-3 [31], [39], as a Wnt activator. As shown in Fig. 1C, 8 h after LiCl treatment 7TFP cells showed a dose-dependent stimulation of Wnt signaling response as indicated by the Firefly luciferase activity. At 50 mM, LiCl elicited a near 1000 fold reporter induction relative to the negative control (DMEM). Therefore, for all subsequent experiments LiCl was used at 50 mM concentration. We further validated the ability of the assay system to identify compounds affecting Wnt signaling using a known inhibitor of Wnt signaling, a synthetic small molecule FH535 obtained from Sigma Chemicals (St. Louis, MO) (standalone validation data not shown). Therefore, the FH535 was used as a reference control for all subsequent experiments as shown in Figs. 2–6.

### Selection of Compounds for their Ability to Inhibit Transcriptional Induction of Wnt Signaling

To identify natural modulators of canonical Wnt signaling and further validate our reporter assay system, we performed pilot and focused screening with five compounds from our laboratory natural product collection. Using greater than 25% inhibition at the highest tested concentration combined with concentration dependent inhibition curve as compared to LiCl positive control, we identified three compounds that downregulated TCF mediated Wnt signaling activation (Fig. 2). Avns 2p and 2f attenuated 30% of the activation at 40 µM concentrations while the same level of attenuation was achieved by 22.24 nM Triptolide. The synthetic FH535 achieved a greater degree of Wnt signaling attenuation at 10 µM concentration. It did not show any activity at nanomolar levels (data not shown). It should be mentioned that no obvious cellular cytopathic effects were observed using a phase contrast microscope during 12 h exposure of cells to all tested compounds. We did not observe significant changes in transcriptional activation levels in response to Avn 2p or Triptolide with prolonged incubations (12 h) as compared to 8 h treatments.

### Attenuation of HeLa Cell Proliferation by Test Agents

Wnt signaling activation has been linked to uncontrolled cell proliferation during carcinogenesis [3]. In our screening experiment using stable Wnt reporter HeLa cells, 2p, 2f and Triptolide suppressed LiCl activated TCF-mediated transcription in a concentration dependent manner. Since deregulated cell proliferation is a hallmark of cancer cells, the antiproliferative effects of the three compounds testing positive in the screening assay (Fig. 2) were determined. Both Triptolide and Avn 2p showed significant, concentration dependent antiproliferative activity (Fig. 3). Avn 2f did not demonstrate antiproliferative activity during 24 h culture, thus, only 2p and Triptolide were studied in subsequent experiments.

### Avn 2p and Triptolide Promoted Cellular *β-catenin* Protein Degradation

Further mechanistic investigation into Wnt signaling perturbation by Triptolide and 2p were carried out. Since Wnt signaling activation that was suppressed by the compounds, requires cellular production of β-catenin and its binding to TCF transcription complex in the nucleus [3], we examined the ability of the compounds to enhance cellular β-catenin protein degradation. Concentration dependent LiCl-induced β-catenin protein degradation was observed in response to 2p and Triptolide in HeLa cells (Fig. 4B). Decreased expression of β-catenin was also observed in FH535-treated cells (Fig. 4B). Notably, endogenous actin expression was not altered in cells treated with 2p or FH535, indicating that the observed reduction of β-catenin was not due to drug toxicity (Fig. 4A).

### Avn 2p and Triptolide Abrogated Nuclear Localization of *β-catenin*

Wnt signaling response is dependent on the nuclear localization of β-catenin where it interacts with TCF/LEF family of transcription factors to activate the Wnt pathway. Therefore, we wanted to confirm if cellular degradation of β-catenin by 2p and Triptolide (Fig. 4) logically leads to reduction in nuclear localization of β-catenin.

As demonstrated in Fig. 5A, nuclear localization of β-catenin was significantly inhibited in HeLa cells treated with both the compounds as compared to untreated cells. Quantitative analysis of the number of cells exhibiting nuclear β-catenin staining out of 100 randomly counted cells from treated or LiCl control further supported our observation (Fig. 5B). Approximately 30% of untreated cells possessed the nuclear translocation of β-catenin in contrast to 14% in 2p- and 8% in Triptolide-treated cells.

### Effect of Avn 2p and Triptolide on Downstream Oncogenic Effector Expression Induced by β-catenin-mediated Aberrant Wnt Signaling

The Wnt signaling pathway leads to the dephosphorylation, stabilization, and nuclear translocation of β-catenin. The stabilized β-catenin binds with the TCF transcription factor complex, leading to the activation of Wnt-responsive genes such as c-Myc. As one of the principal downstream targets of the β-catenin-TCF complex, c-Myc plays a key role in altered cellular metabolism during tumorigenesis [28]. The experiment was designed to quantify the relative amount of transcripts for c-Myc target gene within the total RNA in individual cell batches undergoing dose-dependent treatments. The positive control (treated with LiCl+DMSO) showed the maximum up-regulation of the target gene. The negative control (DMSO only) maintained a constant amount of constitutively expressed gene transcripts and served as a reference baseline. A reduction of mRNA, compared to the positive control, indicated an inhibitory effect of the particular treatment. Expression of β-actin, a constitutively expressed housekeeping gene, served as a quality control step for determining RNA degradation in the assay. The c-Myc mRNA was suppressed by 2p and Triptolide in a concentration-dependent manner (Fig. 6). The half inhibitory concentration of 2p was 80 uM. Triptolide attenuated greater than 80% of the c-Myc relative expression levels at 44.48 nM concentration. The small molecule inhibitor FH535, used as a known reference was effective in the micromolar range.

## Discussion

β-catenin is a multifunctional protein that plays an important role in ontogenesis and oncogenesis. A major consequence of aberrant Wnt signaling is stabilization of β-catenin, and this can be triggered using LiCl-elicitation for *in vitro* studies. Formation of the β-catenin–TCFs complex in the nucleus is a prerequisite for the transcription of Wnt target genes that has been associated with the development of many types of cancers [40]. Dietary natural products that suppress formation of the β-catenin–TCFs complex in the nucleus and subsequently prevent activation of downstream targets, particularly oncogenic targets, might help in prevention and treatment of various cancers. In this study we investigated the effects of five natural compounds in attenuating aberrant activation of Wnt target genes mediated by β-catenin (Fig. 2). These five compounds are well documented for their antioxidant, anti-inflammatory, chemopreventive and other pharmacological properties, and are either widely consumed or well-studied for their biological activities [9], [14], [20], [21], [24]. Three of these compounds, 2p, 2f and 2c are found in oats, a popular cereal consumed worldwide. PEITC is an isothiocyanate found in certain vegetables and is currently in phase 1 clinical trials for lung cancer treatments (NCT ID: NCT00005883, NCT00691132; clinicaltrials.gov). Triptolide is an extremely potent natural product isolated from a Chinese medicinal plant and is known for a broad range of therapeutic activities including cancer treatment. The effects of these compounds on stabilization of β-catenin have never been documented. In this study we also produced a stably transformed HeLa cell-based reporter assay system as a high content, medium throughput, screening tool for long term use. This and similar surrogate reporters of well-characterized biomarker expression offer an effective methodology that can be utilized to carry out large scale screening of natural products for possible mechanisms related to their therapeutic potential. Use of similar robust high content screens could significantly improve the pace of evidence based natural product research and development by offering a comprehensive view of the molecular underpinnings of pathology.

Avns constitute the major phenolic antioxidants present in the oat grain. Preliminary evidence demonstrates antiproliferative effects of Avn-enriched fractions (but not specifically mediated by 2p) [41]. In our experiments, 2p, the least studied among the three major forms of Avns (2p, 2f and 2c), showed the most significant biological activities. Avn 2p modulated upstream events in β-catenin mediated transcriptional activation of Wnt target gene, c-Myc (Fig. 6), and suppressed proliferation of human cervical cancer cells *in vitro* (Fig. 3). Concentration dependent β-catenin degradation in the cytosol was increased by 2p (Fig. 4), which also attenuated nuclear β-catenin accumulation (Fig. 5) and subsequent transcriptional activation of c-Myc (Fig. 6). Since cytosolic degradation of β-catenin precedes the latter events, it is uncertain if Avn 2p is targeting each of these events directly or the downstream effects are a relay of upstream regulation by 2p. The 12 h biological effects of 2p were significant in our conditions at 40–160 µM as compared to a 5–20 µM therapeutic window for FH535, the known β-catenin inhibitor used as a reference positive control. In Human, maximum plasma concentrations of free and conjugated Avn 2p were 112.9 and 374.6 nM, time to reach the C(max) for both doses were 2.30 h, and half times for elimination was 1.75 h [11]. Since 2p can be partially lost during commercial processing of oat cereal or modified by glucuronidation or sulfonation during normal metabolism [16], to achieve required concentrations as part of the diet, fortifications may be necessary. Enhancement of Avn levels through oat breeding programs, agricultural practices [33] or post-harvest processing methods should also be investigated. These novel findings add to the growing knowledge that oat Avns provide a broad range of health benefits that complement the already known health benefits derived from oats. Follow-up research will be necessary to establish efficacy range of Avn 2p for specific forms of cancers and *in vivo* validation of this β-catenin-mediated mechanism of biological activity. Cervical cancer is the second-leading cause of cancer deaths in women worldwide. Use of Avns in cervical cancer prevention may be particularly relevant given that cervical cancer typically develops slowly over many years after initial HPV infection, suggesting that a long-term, convenient prevention strategy such as through diet may be meaningful [5].

Triptolide, the main active component of the traditional Chinese herbal medicine Tripterygium wilfordii Hook F, decreased accumulation of β-catenin in the cytoplasm and attenuated its translocation in to the nucleus, thereby inhibiting transcriptional activation of TCF. Significant pro-apoptotic effects of Triptolide were observed in HeLa cells at nanomolar concentrations as compared to the reference compound FH535 which was effective at micromolar quantities (Fig. 3). Although the effects of Triptolide on Wnt signaling has not been reported, it was shown that Triptolide-induced death of HeLa cells is dependent on AKT inactivation and caspase-9 activation [42]. Interestingly crosstalk exists between phosphatidylinositol 3-kinase (PI3K)/AKT and the Wnt/β-catenin pathways. Inactivation of GSK-3 is a critical event in Wnt/β-catenin signal transduction as GSK-3-mediated phosphorylation of β-catenin targets it for degradation. Our immunoblotting of phosphorylated GSK-3β (Ser9) did not show significant change of phosphorylated GSK-3β abundance in HeLa cell at 12 h treatment by Avn 2p and Triptolide using the concentrations indicated previously (data not shown). Independent of the Wnt-mediated GSK-3 inactivation, the PI3K/AKT pathway also inactivates GSK-3, via direct AKT-mediated phosphorylation of Ser21/9 (for GSK-3α/β) [43]. Therefore, it is possible that the effect of Triptolide on β-catenin degradation is mediated through its effects on AKT inactivation. Further studies in this direction will be undertaken in recent future.

Triptolide also downregulated c-Myc mRNA at nanomolar concentrations in a concentration dependent manner (Fig. 6). The interplay of Wnt/β-catenin and Myc signaling in immature tumors activates a distinct transcriptional program that correlates with tumor aggressiveness in many cancers including in hepatoblastoma [44]. While Triptolide has a divergent therapeutic profile and can perturb multiple signal pathways, its effects on either Wnt/β-catenin or Myc signaling were never reported. It would be interesting to further investigate if the disruption of β-catenin accumulation by this promiscuous phytochemical would lead to abrogation of stem like properties of cancer cells that is often associated with tumor recurrence and aggressiveness. In rat, after oral administration in doses of 0.6 to 2.4 mg/kg, the concentration of Triptolide in plasma reached the maximum within 15 min, and declined rapidly with an half-life from 16.81 to 21.70 min [45]. In HeLa cells however, at nanomolar concentrations, Wnt transcription inhibition by Triptolide has been observed at least for 12 h. Also, current data suggest that the extended Myc network regulates the cellular response to changes in nutrient availability and may be altered in cancer and insulin resistance [46]. Recent epidemiological and clinical evidence points to a link between insulin resistance and cancer. The mechanisms for this association are unknown, but hyperinsulinemia appears to have a role in tumor initiation and progression in insulin-resistant patients [47]. It is interesting to note that in our observations, Triptolide can counter-regulate rate limiting gluconeogenetic enzymes perturbed by insulin resistance under *in vitro* conditions (unpublished results). Whether Triptolide can be targeted to insulin resistant patients with higher risk for cancer development might be a potential line of research.

In conclusion, our study elucidates a novel molecular paradigm mediating previously known pro-apoptotic effects of Triptolide in human cervical cancer cells. This involved disruption of aberrant β-catenin signaling. Our study also demonstrates novel pro-apoptotic effects of Avn 2p, also mediated through abrogation of aberrant β-catenin signaling. Given the extremely low concentrations at which Triptolide is biologically targeting the molecular players discussed above, further investigations are warranted to explore potential use of this compound in clinics for treatment of aggressive cancers involving abnormal β-catenin signaling. Triptolide has been tested on animals and human for various health benefits, hence its therapeutic and toxic concentration windows are already established [48], [49]. Avn 2p, on the other hand, could be a suitable candidate for further investigation as a chemopreventive agent. Interestingly PEITC, with previously validated anticancer effects, did not interact with the Wnt/β-catenin signaling, suggesting a distinct cellular mechanism for its anticancer properties. Hence, phytochemicals present in fruits, vegetables and cereals are suitable candidates for broad-spectrum molecular targeting, specifically when used in complex combinations such as in a diet. If optimized well, these may offer a broad-spectrum, prophylactic and therapeutic potential to overcome the problem of adaptive resistance, particularly in complex diseases such as cancer.
